# *N*-(2,6-Dichloro­phen­yl)benzamide

**DOI:** 10.1107/S160053680800305X

**Published:** 2008-01-30

**Authors:** B. Thimme Gowda, Miroslav Tokarčík, Jozef Kožíšek, B. P. Sowmya, Hartmut Fuess

**Affiliations:** a Department of Chemistry, Mangalore University, Mangalagangotri-574 199, Mangalore, India; b Faculty of Chemical and Food Technology, Slovak Technical University, Radlinského 9, SK-812 37 Bratislava, Slovak Republic; c Institute of Materials Science, Darmstadt University of Technology, Petersenstrasse 23, D-64287, Darmstadt, Germany

## Abstract

The conformation of the N—H and C=O bonds in the structure of the title compound (N26DCPBA), C_13_H_9_Cl_2_NO, are *anti* to each other, similar to that observed in *N*-phenyl­benzamide (NPBA), *N*-(2-chloro­phen­yl)benzamide (N2CPBA), *N*-(2,3-dichloro­phen­yl)benzamide (N23DCPBA) and other benzanilides. The asymmetric unit of N26DCPBA contains two mol­ecules. The bond parameters in N26DCPBA are similar to those in NPBA, N2CPBA, N23DCPBA and other benzanilides. The amide group, –NHCO–, makes a dihedral angle of 30.8 (1)° with the benzoyl ring in the first mol­ecule and 35.1 (2)° in the second mol­ecule of the asymmetric unit. The dihedral angle between the two benzene rings (benzoyl and aniline) is 56.8 (1)° in the first mol­ecule and 59.1 (1)° in the second mol­ecule. N—H⋯O hydrogen bonds give rise to infinite chains running along the *a* axis of the crystal structure.

## Related literature

For related literature, see: Gowda *et al.* (2003 ▶, 2007*a* ▶,*b* ▶,*c* ▶, 2008 ▶).
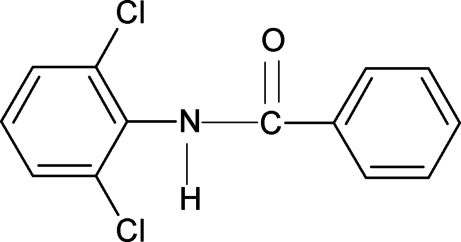


## Experimental

### 

#### Crystal data

C_13_H_9_Cl_2_NO
*M* *_r_* = 266.11
Monoclinic, 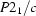

*a* = 10.0431 (2) Å
*b* = 13.7150 (3) Å
*c* = 18.4585 (4) Å
β = 93.623 (2)°
*V* = 2537.41 (9) Å^3^
*Z* = 8
Mo *K*α radiation
μ = 0.49 mm^−1^
*T* = 295 (2) K
0.26 × 0.24 × 0.21 mm

#### Data collection

Oxford Diffraction Xcalibur System diffractometer
Absorption correction: analytical [(Oxford Diffraction, 2007 ▶); analytical numeric absorption correction using a multifaceted crystal model (Clark & Reid, 1995 ▶)] *T*_min_ = 0.882, *T*_max_ = 0.904
54956 measured reflections
4956 independent reflections
3810 reflections with *I* > 2σ(*I*)
*R*_int_ = 0.023

#### Refinement

*R*[*F* ^2^ > 2σ(*F* ^2^)] = 0.043
*wR*(*F* ^2^) = 0.127
*S* = 1.10
4956 reflections
314 parameters
2 restraints
H atoms treated by a mixture of independent and constrained refinement
Δρ_max_ = 0.40 e Å^−3^
Δρ_min_ = −0.35 e Å^−3^

### 

Data collection: *CrysAlis CCD* (Oxford Diffraction, 2007 ▶); cell refinement: *CrysAlis RED* (Oxford Diffraction, 2007 ▶); data reduction: *CrysAlis RED*; program(s) used to solve structure: *SHELXS97* (Sheldrick, 2008 ▶); program(s) used to refine structure: *SHELXL97* (Sheldrick, 2008 ▶); molecular graphics: *ORTEP-3* (Farrugia, 1997 ▶) and *DIAMOND* (Brandenburg, 2002 ▶); software used to prepare material for publication: *SHELXL97*], *PLATON* (Spek, 2003 ▶) and *WinGX* (Farrugia, 1999 ▶).

## Supplementary Material

Crystal structure: contains datablocks I, global. DOI: 10.1107/S160053680800305X/om2209sup1.cif

Structure factors: contains datablocks I. DOI: 10.1107/S160053680800305X/om2209Isup2.hkl

Additional supplementary materials:  crystallographic information; 3D view; checkCIF report

## Figures and Tables

**Table 1:** Hydrogen-bond geometry (Å, °)

*D*—H⋯*A*	*D*—H	H⋯*A*	*D*⋯*A*	*D*—H⋯*A*
N1—H1*N*⋯O2	0.860 (19)	2.09 (2)	2.9035 (18)	158 (2)
N2—H2*N*⋯O1^i^	0.843 (18)	2.060 (19)	2.8831 (18)	165.1 (19)

Symmetry code: (i) 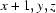.

**Fig. 1.**
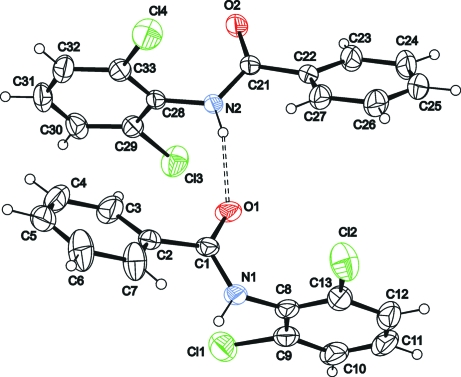
Molecular structure of the title compound showing the atom labelling scheme. Numbers of the C atoms in the second molecule are increased by 20. Displacement ellipsoids are drawn at the 30% probability level. The hydrogen bond N2—H2N···O1 is shown as a dashed line.

**Fig. 2.**
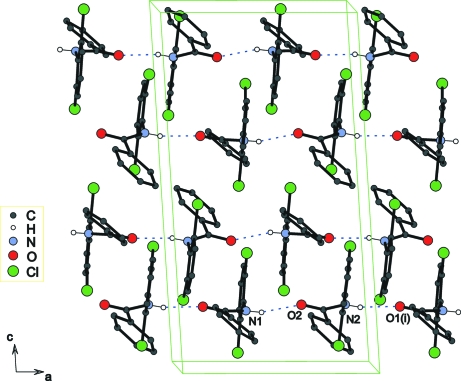
Crystal structure of the title compound viewed down the b axis. One-dimensional chains along the base vector [1 0 0] are formed by hydrogen bonds N1–H1N···O2 and N2–H2N···O1(i). Symmetry code (i): x + 1,y,z. H atoms not involved in hydrogen bonding are omitted.

## supplementary crystallographic information

### Comment

In the present work, the structure of *N*-(2,6-dichlorophenyl)-benzamide (N26DCPBA) has been determined to explore the effect of substituents on the structure of *N*-aromatic amides (Gowda *et al.*, 2003,2007*a*,b,c,2008). The conformation of the N—H and C=O bonds in the structure of N26DCPBA (Fig.1) are anti to each other, similar to that observed in *N*-(phenyl)-benzamide (NPBA)(Gowda *et al.*, 2003), *N*-(2-chlorophenyl)-benzamide (N2CPBA), *N*-(2,3-dichlorophenyl)-benzamide (N23DCPBA) and other benzanilides(Gowda *et al.*, 2007*a*,b,c, 2008). The title compound crystallizes in the space group P21/c, with two molecules in the asymmetric unit. The bond parameters in N26DCPBA are similar to those in NPBA, N2CPBA, N23DCPBA and other benzanilides. The amide group –NHCO– has the dihedral angle of 30.8 (1)° with the benzoyl ring in the first molecule and 35.1 (2)° in the second molecule of the asymmetric unit. The dihedral angle between the two benzene rings (benzoyl and aniline) is 56.8 (1)° in the first molecule and 59.1 (1)° in the second molecule. One-dimensional chains of N26DCPBA along the base vector [1 0 0] formed by hydrogen bonds N1–H1N···O2(i) and N2–H2N···O1 (Table 1) as viewed down the *b* axis is shown in Fig.2. Symmetry code (i): *x* + 1,*y*,*z*.

### Experimental

The title compound was prepared according to the literature method (Gowda *et al.*, 2003). The purity of the compound was checked by determining its melting point. It was characterized by recording its infrared and NMR spectra. Single crystals of the title compound were obtained from an ethanolic solution and used for X-ray diffraction studies at room temperature.

### Refinement

H atoms bonded to C atoms were placed in geometrically calculated positions and subsequently treated as riding with C—H bond distance 0.93 Å. H(N) atoms were visible in the difference map. In the refinement the N—H distance was restrained to 0.86 (4) Å. The *U*_iso_(H) values were set at 1.2 *U*_eq_(C,*N*).

### Crystal data

**Table:**

C_13_H_9_Cl_2_NO	*F*_000_ = 1088
*M**_r_* = 266.11	*D*_x_ = 1.393 Mg m^−^^3^
Monoclinic, *P*2_1_/*c*	Mo *K*α radiation λ = 0.71073 Å
Hall symbol: -P 2ybc	Cell parameters from 24495 reflections
*a* = 10.0431 (2) Å	θ = 3.1–29.5º
*b* = 13.7150 (3) Å	µ = 0.49 mm^−^^1^
*c* = 18.4585 (4) Å	*T* = 295 (2) K
β = 93.623 (2)º	Block, colorless
*V* = 2537.41 (9) Å^3^	0.26 × 0.24 × 0.21 mm
*Z* = 8	

### Data collection

**Table:**

Oxford Diffraction Xcalibur System diffractometer	4956 independent reflections
Radiation source: Enhance (Mo) X-ray Source	3810 reflections with *I* > 2σ(*I*)
Monochromator: graphite	*R*_int_ = 0.023
Detector resolution: 10.434 pixels mm^-1^	θ_max_ = 26.0º
*T* = 295(2) K	θ_min_ = 5.1º
φ scans, and ω scans with κ offsets	*h* = −12→12
Absorption correction: analytical[(Oxford Diffraction, 2007); analytical numeric absorption correction using a multifaceted crystal model (Clark & Reid, 1995)]	*k* = −16→16
*T*_min_ = 0.883, *T*_max_ = 0.904	*l* = −22→22
54956 measured reflections	

### Refinement

**Table:**

Refinement on *F*^2^	Secondary atom site location: difference Fourier map
Least-squares matrix: full	Hydrogen site location: inferred from neighbouring sites
*R*[*F*^2^ > 2σ(*F*^2^)] = 0.043	H atoms treated by a mixture of independent and constrained refinement
*wR*(*F*^2^) = 0.127	*w* = 1/[σ^2^(*F*_o_^2^) + (0.0646*P*)^2^ + 0.4979*P*] where *P* = (*F*_o_^2^ + 2*F*_c_^2^)/3
*S* = 1.10	(Δ/σ)_max_ = 0.001
4956 reflections	Δρ_max_ = 0.40 e Å^−^^3^
314 parameters	Δρ_min_ = −0.35 e Å^−^^3^
2 restraints	Extinction correction: SHELXL97 (Sheldrick, 2008), Fc^*^=kFc[1+0.001xFc^2^λ^3^/sin(2θ)]^-1/4^
Primary atom site location: structure-invariant direct methods	Extinction coefficient: 0.0083 (11)

### Special details

**Table:**

Geometry. All e.s.d.'s (except the e.s.d. in the dihedral angle between two l.s. planes) are estimated using the full covariance matrix. The cell e.s.d.'s are taken into account individually in the estimation of e.s.d.'s in distances, angles and torsion angles; correlations between e.s.d.'s in cell parameters are only used when they are defined by crystal symmetry. An approximate (isotropic) treatment of cell e.s.d.'s is used for estimating e.s.d.'s involving l.s. planes.
Refinement. Refinement of *F*^2^ against ALL reflections. The weighted *R*-factor *wR* and goodness of fit *S* are based on *F*^2^, conventional *R*-factors *R* are based on *F*, with *F* set to zero for negative *F*^2^. The threshold expression of *F*^2^ > σ(*F*^2^) is used only for calculating *R*-factors(gt) *etc*. and is not relevant to the choice of reflections for refinement. *R*-factors based on *F*^2^ are statistically about twice as large as those based on *F*, and *R*- factors based on ALL data will be even larger.

### Fractional atomic coordinates and isotropic or equivalent isotropic displacement parameters (Å^2^)

**Table:**

	*x*	*y*	*z*	*U*_iso_*/*U*_eq_	
C1	0.29163 (15)	0.88930 (13)	0.13557 (10)	0.0468 (4)	
C2	0.31340 (17)	0.99515 (13)	0.12131 (10)	0.0496 (4)	
C3	0.2344 (2)	1.06295 (18)	0.1520 (2)	0.0957 (10)	
H3	0.1718	1.0433	0.184	0.115*	
C4	0.2478 (3)	1.1613 (2)	0.1353 (2)	0.1205 (13)	
H4	0.1942	1.2072	0.1564	0.145*	
C5	0.3392 (3)	1.19053 (19)	0.08819 (19)	0.0957 (10)	
H5	0.3447	1.2559	0.0752	0.115*	
C6	0.4206 (4)	1.1252 (2)	0.06070 (14)	0.0979 (9)	
H6	0.4856	1.1456	0.0304	0.118*	
C7	0.4090 (3)	1.02747 (17)	0.07699 (13)	0.0798 (7)	
H7	0.4667	0.9828	0.0577	0.096*	
C8	0.39430 (16)	0.73180 (13)	0.15565 (12)	0.0555 (5)	
C9	0.41165 (19)	0.70599 (15)	0.22837 (14)	0.0661 (6)	
C10	0.4153 (2)	0.60990 (18)	0.25095 (17)	0.0805 (7)	
H10	0.4284	0.5945	0.3	0.097*	
C11	0.3993 (2)	0.53776 (18)	0.19996 (19)	0.0853 (8)	
H11	0.4011	0.4729	0.2146	0.102*	
C12	0.3805 (2)	0.55993 (17)	0.12743 (18)	0.0822 (8)	
H12	0.3702	0.5102	0.0933	0.099*	
C13	0.3771 (2)	0.65690 (16)	0.10524 (14)	0.0664 (6)	
N1	0.39953 (14)	0.83121 (11)	0.13436 (10)	0.0535 (4)	
H1N	0.4772 (19)	0.8565 (16)	0.1310 (11)	0.064*	
O1	0.18181 (11)	0.85646 (10)	0.14687 (9)	0.0635 (4)	
Cl1	0.42425 (7)	0.79839 (5)	0.29312 (4)	0.0924 (2)	
Cl2	0.35242 (9)	0.68484 (5)	0.01367 (4)	0.1030 (3)	
C21	0.79317 (15)	0.84885 (12)	0.13788 (10)	0.0457 (4)	
C22	0.81608 (17)	0.75379 (13)	0.10084 (11)	0.0505 (4)	
C23	0.7373 (2)	0.67477 (16)	0.11555 (17)	0.0803 (8)	
H23	0.6724	0.6804	0.1491	0.096*	
C24	0.7548 (3)	0.58684 (18)	0.0802 (2)	0.1034 (11)	
H24	0.7023	0.5334	0.0906	0.124*	
C25	0.8487 (3)	0.57822 (19)	0.03020 (18)	0.0922 (9)	
H25	0.8588	0.5193	0.0062	0.111*	
C26	0.9277 (3)	0.65546 (18)	0.01526 (13)	0.0761 (7)	
H26	0.9919	0.6491	−0.0186	0.091*	
C27	0.9124 (2)	0.74372 (15)	0.05076 (11)	0.0588 (5)	
H27	0.9669	0.7963	0.0409	0.071*	
C28	0.89884 (14)	0.99789 (11)	0.18305 (9)	0.0412 (4)	
C29	0.92285 (17)	1.01514 (13)	0.25670 (10)	0.0501 (4)	
C30	0.9290 (2)	1.10850 (16)	0.28488 (12)	0.0622 (5)	
H30	0.9468	1.1184	0.3344	0.075*	
C31	0.9085 (2)	1.18598 (15)	0.23909 (13)	0.0670 (6)	
H31	0.9121	1.249	0.2578	0.08*	
C32	0.8826 (2)	1.17230 (14)	0.16579 (13)	0.0653 (5)	
H32	0.8683	1.2255	0.1351	0.078*	
C33	0.87794 (18)	1.07878 (13)	0.13833 (10)	0.0511 (4)	
N2	0.90279 (14)	0.90255 (10)	0.15368 (8)	0.0450 (4)	
H2N	0.9794 (18)	0.8817 (14)	0.1457 (10)	0.054*	
O2	0.68208 (11)	0.87638 (10)	0.15285 (9)	0.0677 (4)	
Cl3	0.94534 (8)	0.91674 (5)	0.31518 (3)	0.0865 (2)	
Cl4	0.84784 (8)	1.06116 (5)	0.04585 (3)	0.0869 (2)	

### Atomic displacement parameters (Å^2^)

**Table:**

	*U*^11^	*U*^22^	*U*^33^	*U*^12^	*U*^13^	*U*^23^
C1	0.0336 (8)	0.0493 (10)	0.0569 (10)	−0.0039 (7)	−0.0009 (7)	0.0105 (8)
C2	0.0427 (9)	0.0468 (10)	0.0576 (10)	−0.0041 (8)	−0.0106 (8)	0.0080 (8)
C3	0.0452 (11)	0.0592 (14)	0.185 (3)	0.0027 (10)	0.0251 (15)	0.0048 (16)
C4	0.0598 (15)	0.0540 (15)	0.247 (4)	0.0129 (12)	0.004 (2)	−0.001 (2)
C5	0.0892 (19)	0.0524 (14)	0.139 (3)	−0.0148 (13)	−0.0468 (18)	0.0265 (16)
C6	0.163 (3)	0.0603 (15)	0.0716 (15)	−0.0325 (18)	0.0185 (17)	0.0136 (12)
C7	0.125 (2)	0.0535 (12)	0.0637 (13)	−0.0217 (13)	0.0321 (13)	0.0015 (10)
C8	0.0290 (8)	0.0467 (10)	0.0905 (15)	−0.0013 (7)	0.0021 (8)	0.0144 (10)
C9	0.0424 (10)	0.0583 (12)	0.0961 (16)	−0.0013 (9)	−0.0076 (10)	0.0177 (11)
C10	0.0614 (13)	0.0643 (15)	0.114 (2)	0.0050 (11)	−0.0110 (13)	0.0311 (14)
C11	0.0612 (13)	0.0495 (13)	0.145 (3)	0.0099 (10)	0.0081 (14)	0.0293 (15)
C12	0.0664 (14)	0.0485 (12)	0.133 (2)	−0.0020 (10)	0.0192 (15)	−0.0005 (14)
C13	0.0478 (10)	0.0548 (12)	0.0976 (16)	−0.0053 (9)	0.0116 (10)	0.0053 (11)
N1	0.0312 (7)	0.0452 (8)	0.0843 (11)	−0.0067 (6)	0.0043 (7)	0.0129 (8)
O1	0.0320 (6)	0.0573 (8)	0.1014 (11)	−0.0022 (5)	0.0056 (6)	0.0213 (7)
Cl1	0.1026 (5)	0.0785 (4)	0.0927 (5)	−0.0146 (3)	−0.0221 (4)	0.0098 (3)
Cl2	0.1321 (6)	0.0836 (5)	0.0940 (5)	−0.0293 (4)	0.0131 (4)	−0.0028 (4)
C21	0.0333 (8)	0.0438 (9)	0.0596 (10)	0.0012 (7)	0.0003 (7)	−0.0075 (8)
C22	0.0382 (9)	0.0435 (9)	0.0679 (12)	0.0031 (7)	−0.0111 (8)	−0.0117 (8)
C23	0.0445 (11)	0.0524 (12)	0.145 (2)	−0.0056 (9)	0.0107 (12)	−0.0206 (13)
C24	0.0651 (15)	0.0515 (14)	0.192 (3)	−0.0098 (11)	−0.0021 (18)	−0.0332 (17)
C25	0.0757 (16)	0.0631 (15)	0.134 (2)	0.0173 (13)	−0.0220 (16)	−0.0463 (15)
C26	0.0871 (16)	0.0718 (15)	0.0681 (14)	0.0202 (13)	−0.0060 (12)	−0.0257 (11)
C27	0.0664 (12)	0.0531 (11)	0.0563 (11)	0.0077 (9)	−0.0004 (9)	−0.0102 (9)
C28	0.0288 (7)	0.0404 (9)	0.0547 (10)	−0.0010 (6)	0.0049 (7)	−0.0089 (7)
C29	0.0464 (9)	0.0506 (10)	0.0536 (10)	0.0008 (8)	0.0050 (8)	−0.0060 (8)
C30	0.0624 (12)	0.0631 (13)	0.0613 (12)	−0.0083 (10)	0.0060 (10)	−0.0219 (10)
C31	0.0693 (13)	0.0448 (11)	0.0887 (16)	−0.0085 (9)	0.0194 (11)	−0.0236 (11)
C32	0.0701 (13)	0.0409 (10)	0.0862 (16)	−0.0001 (9)	0.0148 (11)	0.0002 (10)
C33	0.0468 (9)	0.0513 (10)	0.0554 (10)	0.0013 (8)	0.0054 (8)	−0.0051 (8)
N2	0.0290 (6)	0.0427 (8)	0.0634 (9)	0.0026 (6)	0.0029 (6)	−0.0147 (6)
O2	0.0316 (6)	0.0583 (8)	0.1137 (12)	−0.0017 (6)	0.0088 (7)	−0.0256 (8)
Cl3	0.1228 (5)	0.0719 (4)	0.0638 (4)	0.0113 (3)	−0.0019 (3)	0.0093 (3)
Cl4	0.1163 (5)	0.0878 (4)	0.0553 (3)	0.0093 (4)	−0.0057 (3)	0.0019 (3)

### Geometric parameters (Å, °)

**Table:**

C1—O1	1.2214 (19)	C21—O2	1.2258 (19)
C1—N1	1.346 (2)	C21—N2	1.341 (2)
C1—C2	1.494 (2)	C21—C22	1.497 (2)
C2—C3	1.368 (3)	C22—C23	1.379 (3)
C2—C7	1.373 (3)	C22—C27	1.386 (3)
C3—C4	1.391 (4)	C23—C24	1.388 (3)
C3—H3	0.93	C23—H23	0.93
C4—C5	1.364 (5)	C24—C25	1.366 (4)
C4—H4	0.93	C24—H24	0.93
C5—C6	1.335 (4)	C25—C26	1.362 (4)
C5—H5	0.93	C25—H25	0.93
C6—C7	1.380 (3)	C26—C27	1.390 (3)
C6—H6	0.93	C26—H26	0.93
C7—H7	0.93	C27—H27	0.93
C8—C9	1.389 (3)	C28—C29	1.386 (2)
C8—C13	1.389 (3)	C28—C33	1.391 (3)
C8—N1	1.421 (2)	C28—N2	1.417 (2)
C9—C10	1.382 (3)	C29—C30	1.382 (3)
C9—Cl1	1.741 (3)	C29—Cl3	1.734 (2)
C10—C11	1.368 (4)	C30—C31	1.365 (3)
C10—H10	0.93	C30—H30	0.93
C11—C12	1.374 (4)	C31—C32	1.374 (3)
C11—H11	0.93	C31—H31	0.93
C12—C13	1.391 (3)	C32—C33	1.379 (3)
C12—H12	0.93	C32—H32	0.93
C13—Cl2	1.736 (3)	C33—Cl4	1.732 (2)
N1—H1N	0.860 (19)	N2—H2N	0.843 (18)
			
O1—C1—N1	121.41 (16)	O2—C21—N2	121.88 (16)
O1—C1—C2	122.15 (16)	O2—C21—C22	122.66 (15)
N1—C1—C2	116.43 (14)	N2—C21—C22	115.46 (14)
C3—C2—C7	118.2 (2)	C23—C22—C27	119.09 (18)
C3—C2—C1	119.51 (18)	C23—C22—C21	119.21 (18)
C7—C2—C1	122.29 (18)	C27—C22—C21	121.68 (16)
C2—C3—C4	120.1 (3)	C22—C23—C24	119.9 (2)
C2—C3—H3	120	C22—C23—H23	120
C4—C3—H3	120	C24—C23—H23	120
C5—C4—C3	120.3 (3)	C25—C24—C23	120.4 (2)
C5—C4—H4	119.9	C25—C24—H24	119.8
C3—C4—H4	119.9	C23—C24—H24	119.8
C6—C5—C4	119.9 (2)	C26—C25—C24	120.4 (2)
C6—C5—H5	120.1	C26—C25—H25	119.8
C4—C5—H5	120.1	C24—C25—H25	119.8
C5—C6—C7	120.5 (3)	C25—C26—C27	119.9 (2)
C5—C6—H6	119.8	C25—C26—H26	120.1
C7—C6—H6	119.8	C27—C26—H26	120.1
C2—C7—C6	121.0 (3)	C22—C27—C26	120.3 (2)
C2—C7—H7	119.5	C22—C27—H27	119.9
C6—C7—H7	119.5	C26—C27—H27	119.9
C9—C8—C13	117.49 (18)	C29—C28—C33	117.15 (15)
C9—C8—N1	120.5 (2)	C29—C28—N2	121.70 (16)
C13—C8—N1	122.0 (2)	C33—C28—N2	121.06 (16)
C10—C9—C8	122.3 (2)	C30—C29—C28	121.88 (18)
C10—C9—Cl1	119.2 (2)	C30—C29—Cl3	119.07 (15)
C8—C9—Cl1	118.51 (16)	C28—C29—Cl3	119.05 (13)
C11—C10—C9	118.8 (3)	C31—C30—C29	119.13 (19)
C11—C10—H10	120.6	C31—C30—H30	120.4
C9—C10—H10	120.6	C29—C30—H30	120.4
C10—C11—C12	120.9 (2)	C30—C31—C32	120.99 (18)
C10—C11—H11	119.6	C30—C31—H31	119.5
C12—C11—H11	119.6	C32—C31—H31	119.5
C11—C12—C13	119.8 (3)	C31—C32—C33	119.3 (2)
C11—C12—H12	120.1	C31—C32—H32	120.4
C13—C12—H12	120.1	C33—C32—H32	120.4
C8—C13—C12	120.7 (2)	C32—C33—C28	121.57 (18)
C8—C13—Cl2	119.53 (16)	C32—C33—Cl4	119.47 (16)
C12—C13—Cl2	119.8 (2)	C28—C33—Cl4	118.95 (14)
C1—N1—C8	121.29 (14)	C21—N2—C28	123.19 (14)
C1—N1—H1N	119.9 (14)	C21—N2—H2N	121.5 (13)
C8—N1—H1N	117.2 (15)	C28—N2—H2N	115.3 (13)
			
O1—C1—C2—C3	30.4 (3)	O2—C21—C22—C23	−34.2 (3)
N1—C1—C2—C3	−150.5 (2)	N2—C21—C22—C23	145.9 (2)
O1—C1—C2—C7	−148.3 (2)	O2—C21—C22—C27	144.2 (2)
N1—C1—C2—C7	30.8 (3)	N2—C21—C22—C27	−35.7 (3)
C7—C2—C3—C4	3.0 (4)	C27—C22—C23—C24	−0.2 (4)
C1—C2—C3—C4	−175.7 (3)	C21—C22—C23—C24	178.3 (2)
C2—C3—C4—C5	0.2 (5)	C22—C23—C24—C25	−0.8 (4)
C3—C4—C5—C6	−3.3 (5)	C23—C24—C25—C26	1.0 (5)
C4—C5—C6—C7	3.0 (5)	C24—C25—C26—C27	−0.4 (4)
C3—C2—C7—C6	−3.3 (4)	C23—C22—C27—C26	0.9 (3)
C1—C2—C7—C6	175.4 (2)	C21—C22—C27—C26	−177.54 (18)
C5—C6—C7—C2	0.4 (4)	C25—C26—C27—C22	−0.6 (3)
C13—C8—C9—C10	1.5 (3)	C33—C28—C29—C30	−1.4 (3)
N1—C8—C9—C10	−176.03 (18)	N2—C28—C29—C30	175.06 (16)
C13—C8—C9—Cl1	−176.52 (14)	C33—C28—C29—Cl3	178.37 (13)
N1—C8—C9—Cl1	5.9 (2)	N2—C28—C29—Cl3	−5.1 (2)
C8—C9—C10—C11	−1.0 (3)	C28—C29—C30—C31	1.2 (3)
Cl1—C9—C10—C11	177.08 (18)	Cl3—C29—C30—C31	−178.63 (16)
C9—C10—C11—C12	0.3 (4)	C29—C30—C31—C32	−0.3 (3)
C10—C11—C12—C13	−0.3 (4)	C30—C31—C32—C33	−0.3 (3)
C9—C8—C13—C12	−1.5 (3)	C31—C32—C33—C28	0.0 (3)
N1—C8—C13—C12	176.04 (18)	C31—C32—C33—Cl4	−179.03 (16)
C9—C8—C13—Cl2	179.02 (14)	C29—C28—C33—C32	0.8 (3)
N1—C8—C13—Cl2	−3.4 (2)	N2—C28—C33—C32	−175.69 (17)
C11—C12—C13—C8	0.9 (3)	C29—C28—C33—Cl4	179.88 (12)
C11—C12—C13—Cl2	−179.60 (17)	N2—C28—C33—Cl4	3.4 (2)
O1—C1—N1—C8	−8.5 (3)	O2—C21—N2—C28	−5.4 (3)
C2—C1—N1—C8	172.35 (18)	C22—C21—N2—C28	174.49 (16)
C9—C8—N1—C1	−84.0 (2)	C29—C28—N2—C21	98.8 (2)
C13—C8—N1—C1	98.5 (2)	C33—C28—N2—C21	−84.8 (2)

### Hydrogen-bond geometry (Å, °)

**Table:**

*D*—H···*A*	*D*—H	H···*A*	*D*···*A*	*D*—H···*A*
N1—H1N···O2	0.860 (19)	2.09 (2)	2.9035 (18)	158 (2)
N2—H2N···O1^i^	0.843 (18)	2.060 (19)	2.8831 (18)	165.1 (19)

Symmetry codes: (i) *x*+1, *y*, *z*.
